# Occupational cancer claims in Korea from 2010 to 2016

**DOI:** 10.1186/s40557-018-0272-6

**Published:** 2018-11-01

**Authors:** Kyungjoon Lee, Sujin Lee, Jeehee Min, Inah Kim

**Affiliations:** 1 Department of Occupational and Environmental Medicine, Hanyang University Hospital, 222-1 Wangsimni-ro, Seongdong-gu, Seoul, 04763 Republic of Korea; 2 Department of Occupational and Environmental Medicine, College of Medicine, Hanyang University, 222 Wangsimni-ro, Seongdong-gu, Seoul, 04763 Republic of Korea

**Keywords:** Occupational cancer, Approval rate, Claims data

## Abstract

**Background:**

Research on carcinogens causing occupational cancer has been updated. Further, social interest in occupational cancer has increased. In addition, the standard for recognizing cancer as a work-related disease has also been revised. The present study aims to describe the distribution of occupational cancer claims or its approval rate and their association with work-related variables.

**Methods:**

We analyzed 1299 claim cases for occupational cancer from 2010 to 2016 provided by the Korea Workers’ Compensation and Welfare Service (KCOMWEL). The status of approval rate was shown by year, sex, industry, occupation, age of diagnosis, duration from employment to diagnosis, and cancer site.

**Results:**

The approval rate was 39.0% from 2010 to 2016 and tended to increase annually since 2011. Both the number of claims and the approval rate were higher in men. Mining and quarrying showed the highest approval rate (78.4%). The approval rates by age of diagnosis and duration from employment to diagnosis increased as the time periods increased. Respiratory organ had the highest number of claims and the highest approval rate by cancer site.

**Conclusions:**

The approval rate of occupational cancer has shown an increasing trend since 2011. The increase of occupational carcinogens and cancer sites and the improvement of social awareness about occupational cancer could have resulted in this trend. The present study provides unique, and the latest and most accurate findings on occupational cancer data of recent 7 years that could be helpful to researchers or policy makers on occupational cancer.

## Background

Occupational cancer is developed by occupational exposure to carcinogens or if the risk of cancer is higher during specific occupational situations [1]. Because cancer is a latent disease and evidence on workers’ exposure to occupational carcinogens is insufficient, it is difficult to investigate its work-relatedness or classify it as an occupational disease.

According to Article 34 of the Enforcement Decree of the Industrial Accident Compensation Insurance (IACI) Act, 3 criteria need to be met for the recognition of work-related diseases in Korea: 1) history of carcinogen exposure; 2) determination of the cumulative exposure level and latent period, which is the period between first exposure to causative agent and diagnosis of cancer; and 3) consideration of medically recognized causal relationships [2]. However, the act did not adequately reflect the newly found occupational risk factors, as the industrial structure and working environment have changed. In addition, accumulation of epidemiologic findings and the development of diagnostic techniques have also identified causal associations between risk factors and diseases. In this background, the number of occupational carcinogens has expanded from 9 to 23 species, and the number of work-related cancers has expanded from 9 to 21 types since July 2013 [3].

A case of mesothelioma was first officially approved as occupational cancer by the Korea Workers’ Compensation and Welfare Service (KCOMWEL) in 1993 in Korea [4]. From 1992 to 2000, only 35 cases out of 417 claims were approved, based on the epidemiologic investigation by the Occupational Safety and Health Research Institute (OSHRI). The KCOMWEL approved 447 cases as occupational cancer out of 2328 claims from 2000 to 2011 [5]. After this period, no study was performed on claimed cases of occupational cancer, especially about detailed information such as approval rate or target organ of cancer that contain important meaning regarding occupational cancer.

Therefore, the present study aims to describe the distribution or approval rate of occupational cancer claims according to work-related variables, and to investigate the association between them.

## Methods

### Study subjects

We analyzed claims data for occupational cancer from 2010 to 2016 provided by the KCOMWEL. Since the data included not only cancer cases but also cases of other diseases (such as cardiovascular or musculoskeletal disease), we selected cancer cases through the following process.

By including cases classified by “code 19 (malignant neoplasm and occupational cancer)” of the main category number and cases not classified by “code 19” but described with words that mean cancer (such as malignant, cancer, tumor, metastasis, etc.), we first selected 1497 cases. Of course, they were classified as cancer on the KCD (Korean Standard Classification of Diseases) code at the same time. In particular, multiple myeloma, myelofibrosis, myelodysplastic syndrome, aplastic anemia and essential thrombocytosis were included and classified by other lymphohematopoietic diseases.

Subsequently, we excluded duplicate data. However, if cases were re-claimed on a different date with the same diagnosis, we included the earlier case if it was finally disapproved and the recent case if it was finally approved. We excluded 9 cases by this method.

We also excluded misclassified cases not described with words meaning cancer. However, of these cases, we included those that were evaluated as being caused by cancer, such as cauda equina syndrome caused by cancer of the spinal cord. We excluded 162 cases by this method.

Other cases that were classified as cancer but the exact target organ or diagnostic code was not found were identified individually by the KCOMWEL. In other words, we included only the cases in which both the target organ and the diagnostic code were confirmed. Although cases of metastatic cancer were excluded in accordance with these criteria, it will be described in the discussion section as it requires some explanation. We excluded 27 cases by this method.

Finally, we included 1299 cases for analysis by this process. Of the 198 excluded cases, 151 cases (76.3%) were from 2010 to 2013. From 2014 data, errors in data classification have been reduced relative to previous years.

### Work-related variables

Data provided by the KCOMWEL included various data on claims, instances, job characteristics, type of industry or occupation, date of claim, date of diagnosis, status of approval. Approval rate was calculated by dividing the number of approval cases by the number of total claims.

## Results

We analyzed 1299 claim cases for occupational cancer of recent 7 years from 2010 to 2016. The annual approval status is shown in Table 1. The approval rate was 39.0% and tended to increase annually since 2011. The number of claims was higher in men (87.6%) than in women (12.3%), and the approval rate was higher in men too (42.4% for men and 15% for women) (Table 2).

**Table 1:** Distribution of occupational cancer claims and approvals by year

Year	Total claims		Approvals	
	Cases	%	Cases	%
2010	167	12.9	57	11.7
2011	152	11.7	37	7.3
2012	170	13.1	55	10.8
2013	190	14.6	69	13.6
2014	214	16.5	77	15.2
2015	187	14.4	87	17.2
2016	219	16.9	125	24.7
Total	1299	100.0	507	100.0

This is the result of analyzing 1299 claim cases for occupational cancer from 2010 to 2016 provided by the Korea Workers’ Compensation and Welfare Service (KCOMWEL)

**Table 2:** Distribution of occupational cancer claims and approvals by sex

Sex	Total claims		Approvals	
	Cases	%	Cases	%
Male	1138	87.6	483	95.3
Female	160	12.3	24	4.7
Unclassified	1	0.1		
Total	1299	100.0	507	100.0

This is the result of analyzing 1299 claim cases for occupational cancer from 2010 to 2016 provided by the Korea Workers’ Compensation and Welfare Service (KCOMWEL)

The approval status by industry and occupation is shown in Table 3. Classification of industry type was based on the tax rate schedule of IACI prescribed by the Ministry of Employment and Labor. More than half of the total claims were made by manufacturing (51.1%). Mining and quarrying showed the highest approval rate (78.4%). There was no claim by fishing. Classification of occupation type followed the method of the 6th Korean Standard Classification of Occupations, established based on the International Standard Classification of Occupations. The number of claims was highest for elementary workers (30.5%), and the approval rate was highest for craft and related trades workers (57.7%), while skilled agricultural, forestry, and fishery workers had few claims.

**Table 3:** Distribution of occupational cancer claims and approvals by industry and occupation

	Total Claims		Approvals	
	Cases	%	Cases	%
Industry				
Financial and insurance activities	23	1.8	8	1.6
Mining and quarrying	185	14.2	145	28.6
Manufacturing	664	51.1	242	47.7
Electricity, gas, steam and water supply	4	0.3	1	0.2
Construction	73	5.6	39	7.7
Transportation, storage and communication	62	4.8	17	3.4
Forestry	3	0.2		
Agriculture	4	0.3	1	0.2
Other business	276	21.2	53	10.5
Unclassified	5	0.4	1	0.2
Occupation				
Managers	197	15.2	43	8.5
Professionals and related Workers	87	6.7	21	4.1
Clerks	79	6.1	16	3.2
Service workers	14	1.1		
Sales workers	19	1.5	2	0.4
Skilled agricultural, forestry and fishery workers	2	0.2	2	0.4
Craft and related trades workers	239	18.4	138	27.2
Equipment, machine operating and assembling workers	263	20.2	80	15.8
Elementary workers	396	30.5	205	40.4
Economically dependent workers	3	0.2		
Total	1299	100.0	507	100.0

This is the result of analyzing 1299 claim cases for occupational cancer from 2010 to 2016 provided by the Korea Workers’ Compensation and Welfare Service (KCOMWEL)

The distribution of approval status by age of diagnosis and duration from employment to diagnosis (year) is shown in Table 4. The average age of diagnosis was 53.0 years (standard deviation [SD] 12.5). The average of the duration from employment to diagnosis was 14.9 years (SD 12.8), which refers to the working period at the workplace where the claim was made.

**Table 4:** Distribution of occupational cancer claims and approvals by age of diagnosis and duration from employment to diagnosis

	Total Claims		Approvals	
	Cases	%	Cases	%
Age of diagnosis (years) ^a^				
< 20	2	0.2		
20–29	45	3.5	4	0. 8
30–39	136	10.5	27	5.3
40–49	304	23.4	75	14.8
50–59	444	34.2	158	31.2
60–69	232	17.9	141	27.8
70–79	115	8.9	86	17.0
> 80	21	1.6	16	3.2
Duration from employment to diagnosis (years) ^b^				
< 1	136	10.5	32	6.3
> 1, < 5	242	18.6	52	10.3
> 5, < 10	192	14.8	51	10.1
> 10, < 15	133	10.2	47	9.1
> 15, < 20	122	9.4	52	10.3
> 20, < 25	160	12.3	65	12.8
> 25, < 30	127	9.8	78	15.4
> 30, < 35	80	6.2	51	10.1
> 35, < 40	48	3.7	34	6.7
> 40	59	4.5	45	8.9
Total	1299	100.0	507	100.0

This is the result of analyzing 1299 claim cases for occupational cancer from 2010 to 2016 provided by the Korea Workers’ Compensation and Welfare Service (KCOMWEL)

^a^Average was 53.0 years (Standard deviation 12.5); ^b^Average was 14.9 years (Standard deviation 12.8)

The approval status by cancer site, or target organ of cancer, is possibly the most interesting topic (Table 5). The classification of cancer site is based on that of the International Agency for Research on Cancer (IARC) [6]. Respiratory organ (mostly lung cancer) had the highest number of claims and the highest rate of approval. Further, the approval rate of malignant mesothelioma (pleura and peritoneum) was the highest in the unit of tissue.

**Table 5:** Distribution of occupational cancer claims and approvals by cancer site

Cancer site	Total claims		Approvals			Total claims		Approvals	
	Cases	%	Cases	%		Cases	%	Cases	%
Lip, oral cavity, and pharynx					Breast and female genital organs				
Lip	4	21.1			Breast	23	85.2	2	66.7
Tonsil	5	26.3			Uterine cervix	2	7.4	1	33.3
Nasopharynx	10	52.6	1	100	Ovary	2	7.4		
Total	19	1.5	1	0.2	Total	27	2.1	3	0.6
Digestive organs					Male genital organs				
Oesophagus	5	1.8			Prostate	3	75.0		
Stomach	73	26.6	5	25.0	Testis	1	25.0		
Colon and rectum	33	12.0	2	10.0	Total	4	0.3		
Liver and bile duct	137	50.0	12	60.0	Urinary tract				
Gall bladder	5	1.8	1	5.0	Kidney	12	60.0	2	40.0
Pancreas	14	5.1			Urinary bladder	8	40.0	3	60.0
Other cancers	7	2.6			Total	20	1.5	5	1.0
Total	274	21.1	20	3.9	Brain, and central nervous system				
Respiratory organs					Brain and central nervous system	45	100.0	1	100.0
Nasal cavity and paranasal sinus	5	0.8	2	0.6	Total	45	3.5	1	0.2
Larynx	7	1.2	2	0.6	Endocrine glands				
Lung	581	98.0	355	98.8	Thyroid	28	100.0		
Total	593	45.7	359	70.8	Total	28	2.2		
Bone, skin, and mesothelium, endothelium, and soft tissue					Lymphoid, hematopoietic, and related tissue				
Bone	2	2.4			Leukaemia	102	49.5	38	52.1
Skin (melanoma)	4	4.8	1	2.2	Lymphoma (non hodgkin)	51	24.8	15	20.5
Skin (other malignant neoplasms)	3	3.6			Lymphoma (hodgkin)	3	1.5		
Mesothelium (Pleura and peritoneum)	62	74.7	44	97.8	Other lymphohematopoietic diseases	50	24.3	20	27.4
Other cancers	12	14.5			Total	206	15.9	73	14.4
Total	83	6.4	45	8.9	TOTAL	1299	100.0	507	100.0

This is the result of analyzing 1299 claim cases for occupational cancer from 2010 to 2016 provided by the Korea Workers’ Compensation and Welfare Service (KCOMWEL)

## Discussion

Our analysis of 1299 claim cases of occupational cancer during the recent 7 years from 2010 to 2016 in Korea showed that there were about 185 claims per year on average. The approval rate was 39.0% from 2010 to 2016 and tended to increase annually since 2011. Both the number of claims and the approval rate were higher in men. Mining and quarrying showed the highest approval rate by industry. The approval rates by age of diagnosis and duration from employment to diagnosis increased as the time periods increased. Respiratory organ had the highest number of claims and the highest approval rate by cancer site. As mentioned above, the approval status was determined by performing several procedures for being recognized as an occupational cancer. In 2010, 4 cases had been approved by the KCOMWEL as the first decision but were finally confirmed as disapproved, and 31 cases had the opposite change (initially disapproved but later approved). There has been no change in the approval status of any case since 2011.

The approval rate has shown an increasing trend since 2011. The increase of occupational carcinogens and cancer sites and the improvement of social awareness about occupational cancer could have resulted in this trend [3, 5]. Unlike in the past, when various clinical specialists participated in the evaluation of approval, professionalism and consistency of judgment have been enhanced by the establishment of a professional cancer decision-making committee composed of oncology specialists, occupational and environmental medicine specialists, and legal experts since 2013. Moreover, the number of people with occupational diseases and illnesses (including fatality) has increased approximately by 8% during the same period (from 7247 in 2011 to 7876 in 2016) [7].

In 2014, the number of newly diagnosed patients for cancer of all sites was 112,882 for men (52.0%) and 104,175 for women (48.0%) in Korea [8]. Compared to these relatively minor gender differences, our results showed a significantly high frequency of claims in males. This probably reflects the fact that a high proportion of men have worked in workplaces that are considered to have a higher risk of exposure to occupational carcinogens. For instance, about 2.98 million men (75.9%) and 0.95 million women (24.1%) were employed in the manufacturing industry per year from 2012 to 2016 where more than half of the total claims were made [9].

Several studies have estimated the occupational attributable fraction of cancer. Nurminen and Karjalainen estimated that work-related cancers accounted for about 8% (14% for men, 2% for women) of all malignancies in Finland [10]. Steenland et al. estimated that between 2.4 and 4.8% (3.3–7.3% for men, 0.8–1.0% for women) of cancer deaths were caused by occupational carcinogens in the United States [11]. Rushton et al. estimated the cancer cases attributable to occupational carcinogens to be 5.3% (8.2% for men, 2.3% for women) in the United Kingdom [12]. Kim et al. estimated that about 1.1% of all cancer cases and 1.7% of all cancer deaths in 2005 were caused by occupational carcinogens in Korea [13]. When understanding the differences of attributable fractions across countries, various workplace variables including history of industrial development should be considered. On the other hand, the number of claims or approval rate would be strongly affected by social security system, especially by health insurance system. Because of these differences, it is not meaningful to compare the cases of the claim data with the estimated cases of attributable fractions. Nevertheless, the number of claim seems too small. Among the various problems that could have caused this gap, workers’ responsibility for proving work-relatedness would have played a role. Since this burden on workers has been relieved since 2017, the approval rate would probably continue to rise.

According to Canadian studies, the construction industry accounted for most compensation cases for occupational cancer, especially lung cancer and mesothelioma, along with manufacturing and mining [14, 15]. It was also reported that most workers employed in construction-related industries were estimated to be exposed to asbestos these days [16]. They were also known to be exposed to high levels of solar ultraviolet radiation, which can cause skin cancer [17]. Even after considering the differences in the working environment in Canada, the number of claims and approvals in the Korean construction industry seems low. The current system for classifying IACI policyholders, which does not reflect the characteristics of the construction process of complex works, might have caused this difference.

The population of farm households was 2.50 million, accounting for 4.9% of the total Korean population, in 2016 [18]. The number of agricultural workers was 1.37 million, accounting for 5.1% of total employees, while manufacturing workers comprised 16.7% in the same period [19]. Although agricultural workers have been known to have an increased risk for several cancers, such as hematopoietic cancers due to pesticide exposure or lip cancer and melanoma due to sun exposure [20], just 1 case was approved. As self-employed farmers and small family farmers have been excluded from IACI, the rate of farmers covered by IACI has been low, at 3.0% in 2010 and 5.1% in 2015 [7, 21]. In addition to the agriculture industry, it is necessary to conduct a survey on all types of industries with a relatively small number of claims compared to the population.

The number of claims by age of diagnosis tended to increase until 50s but decreased with age from 60s. A little differently, the approval rate increased with age. The approval rate also increased as the duration from employment to diagnosis increased. Based on cancer latency, the risk-exposure period was defined for the relevant period for cancer development [22]. For the deficiency of data regarding this topic, latency has been assumed; solid tumor for 10 to 50 years or lymphohematopoietic cancers for 0 to 20 years. To minimize the limitations of these assumptions used frequently in studies on occupational cancer, it is necessary to precisely manage industrial accidents statistics.

Lung cancer had the highest number of claims and the 2nd highest rate of approval after malignant mesothelioma. It was reported that 210 cases of occupational lung cancer were approved in Korean IACI subscribers from 1994 to 2011 [23]. Kim et al. reported the population attributable fraction of lung cancer as 3.7% by reflecting Korean studies [13], and the 5-year prevalence of lung cancer was about 37 thousands in 2011 (the number of cancer survivors among patients diagnosed with lung cancer for 5 years before the first day of 2012) [24]. About 11.7% of all lung cancers were estimated to be definite and probable occupational lung cancer by a construction surveillance system [25]. However, there was an obvious difference between results estimated through research and those actually recognized. This difference can also be found in Canada, where 402 claims for occupational lung cancer death was accepted and compensated between 2006 and 2010, which accounted for 0.6% of total lung cancers [15]. The number of cases recognized as occupational lung cancer has been gradually increasing because of the broadening of benefit eligibility covered by IACI and as a result of improvements in the national monitoring system. In addition, as lung cancer recognized as a complication of pneumoconiosis was not included in this study, the number of recognized occupational lung cancer would be more. Nonetheless, it is necessary to build a sustainable and precise surveillance system for understanding the exact situation and implementing a preventive policy.

Lymphohematopoietic cancer accounts for a minor part in Korea. The number of newly diagnosed patients was about 3877 for non-Hodgkin lymphoma (NHL, 1.76% of all cancers) and 2984 for leukemia (1.36% of all cancers) per year on average from 2010 to 2016 [24]. Driscoll et al. reported that about 2% of leukemia cases was attributable to occupational causes [26]. Kim et al. estimated the population attributable fraction as 3.4% for leukemia and 1.8% for NHL using relative risks reported in Korean studies [13]. Although our results still showed a low number of claims than the estimates, it showed a high proportion (15.9% of all claims) in comparison to other cancer sites, considering the relatively low incidence. This might be because of the public concern over reports of leukemia and NHL among workers in the semiconductor industry [27]. Although the occupational exposure level for benzene, the major occupational carcinogen for lymphohematopoietic cancers, has been lowered, more effort to monitor and control the other exposure routes for carcinogens is needed.

Unfortunately, information necessary for evaluating the claims was not included in the data provided by the KCOMWEL. In particular, it was so regrettable that there was no data on the history of exposure to carcinogens. At least some information on the list of exposed carcinogens should be included. In addition, this data may not contain all claims data, when considering several errors (for instance, case of noise induced hearing loss was classified by cancer of labia minora, while both terms share the same 2 syllables in Korean). Nevertheless, we are confident that the results of the present study are the most accurate and latest findings on the data of recent 7 years. We also hope that our results can be applied and used in research or policies to prevent and compensate for workers’ occupational cancer. Also, if there are enough cases for each type of cancer, similar analysis should be performed according to the the type of cancer in the future studies.

## Conclusion

We described the approval rate of occupational cancer in Korea from 2010 to 2016 analyzing the data provided by the KOMWEL. The approval rate was mainly described by year of approval, sex, type of industry or occupation, age of diagnosis, duration from employment to diagnosis, and cancer site. The present study would provide unique, and the latest and most accurate findings on occupational cancer data of recent 7 years.

## Abbreviations

IACI: Industrial Accident Compensation Insurance
IARC: International Agency for Research on Cancer
KCOMWEL: Korea Workers’ Compensation and Welfare Service
OSHRI: Occupational Safety and Health Research Institute

## References

[1] Ahn YS (2011). Occupational cancer update. Korean J Occup Environ Med.

[2] Ministry of Employment and Labor. The enforcement decree under Industrial Accident Compensation Insurance (IACI) Act [Presidential Decree No.22101, 26. Mar, 2010, Partial Amendment].

[3] Song J, Woo KH KY. Guidelines for recognition of occupational cancers in Korea: the results of scientific review by Korean society of. Occupational and environmental medicine (2013-2016). Ann Occup Env Med. 2018;30:12. 10.1186/s40557-018-0223-2 PMC5809845 29456866

[4] Park MI, Choi JS, Choi HM, Jang TI, Moon IH, Kim JH, Jang TW, Lee DH, Jung MHKS (1995). A case of diffuse malignant pleural mesothelioma with occupational asbestos exposure. Korean J Med.

[5] Kim I, Kim EA, Kim JY (2014). Compensation for occupational cancer. J Korean Med Sci.

[6] International Agency for Research on Cancer (1994). List of classifications by cancer sites with sufficient or limited evidence in humans, volumes 1 to 120. International Agency for Research on Cancer.

[7] Ministry of Employment and Labor. Status of industrial accident. [Accessed on 18 March 2018]. Available at: http://www.index.go.kr/potal/main/EachDtlPageDetail.do?idx_cd=1514.

[8] Jung KW, Won YJ, Oh CM, Kong HJ, Lee DH, Lee KH (2017). Cancer statistics in Korea: incidence, mortality, survival, and prevalence in 2014. Cancer Res Treat.

[9] Ministry of Employment and Labor. Employment of establishment. [Accessed on 18 March 2018]. Available at: http://laborstat.molab.go.kr/newOut/renewal/menu06/menu06_search.jsp.

[10] Nurminen M, Karjalainen A (2001). Epidemiologic estimate of the proportion of fatalities related to occupational factors in Finland. Scand J Work Environ Health.

[11] Steenland K, Burnett C, Lalich N, Ward E, Hurrell J (2003). Dying for work: the magnitude of us mortality from selected causes of death associated with occupation. Am J Ind Med.

[12] Rushton Lesley, Hutchings Sally J, Fortunato Lea, Young Charlotte, Evans Gareth S, Brown Terry, Bevan Ruth, Slack Rebecca, Holmes Phillip, Bagga Sanjeev, Cherrie John W, Van Tongeren Martie (2012). Occupational cancer burden in Great Britain. British Journal of Cancer.

[13] Kim Eun-A, Lee Hye-Eun, Kang Seong-Kyu (2010). Occupational Burden of Cancer in Korea. Safety and Health at Work.

[14] Pichora EC, Payne JI (2007). Trends and characteristics of compensated occupational cancer in Ontario, Canada, 1937-2003. Am J Ind Med.

[15] Del Bianco A., Demers P. A. (2013). Trends in compensation for deaths from occupational cancer in Canada: a descriptive study. CMAJ Open.

[16] Occupational exposure estimates: asbestos. Vancouver (BC): Carex Canada; 2012. [Accessed on 18 March 2018]. Available at: www.carexcanada.ca/en/asbestos/occupational_exposure_estimates/phase_2/.

[17] Peters Cheryl E., Demers Paul A., Kalia Sunil, Nicol Anne-Marie, Koehoorn Mieke W. (2016). Levels of Occupational Exposure to Solar Ultraviolet Radiation in Vancouver, Canada. Annals of Occupational Hygiene.

[18] Statistics Korea (2016). Agriculture, forestry and fishery census report.

[19] Statistics Korea (2016). Regional employment survey.

[20] Kachuri L, Harris MA, MacLeod JS, Tjepkema M, Peters PA, Demers PA (2017). Cancer risks in a population-based study of 70,570 agricultural workers: results from the Canadian census health and environment cohort (CanCHEC). BMC Cancer.

[21] Ministry for Food, Agriculture, Forestry and fisheries. Food and rural affairs 2016. [Accessed on 18 March 2018]. Available at: www.mafra.go.kr/bbs/mafra/131/189990/download.do.

[22] Hutchings Sally J, Rushton Lesley (2012). Occupational cancer in Britain. British Journal of Cancer.

[23] Occupational Cancer Review Report by Risk factors in 2013; Asbestos, benzene, painting, Trichlorethylene [translated by Lee KJ]. Korean Society of Occupational and Environmental Medicine.

[24] Ministry of Health & Welfare. Cancer statistic. [Accessed on 18 March 2018]. Available at: http://kosis.kr/statHtml/statHtml.do?orgId=117&tblId=DT_117N_A00122&conn_path=I2.

[25] Leem Jong-Han, Kim Hwan-Cheol, Ryu Jeong-Seon, Won Jong Uk, Moon Jai Dong, Kim Young-Chul, Koh Sang Baek, Yong Suk Joong, Kim Soo Geun, Park Jae Yong, Kim Inah, Kim Jung Il, Kim Jung Won, Lee Eui-cheol, Kim Hyoung-Ryoul, Kim Dae-Hwan, Kang Dong Mug, Hong Yun-Chul (2010). Occupational Lung Cancer Surveillance in South Korea, 2006-2009. Safety and Health at Work.

[26] Driscoll Timothy, Nelson Deborah Imel, Steenland Kyle, Leigh James, Concha-Barrientos Marisol, Fingerhut Marilyn, Prüss-Üstün Annette (2005). The global burden of disease due to occupational carcinogens. American Journal of Industrial Medicine.

[27] Kim EA, Lee WJ, Son M, Kang SK (2010). Occupational lymphohematopoietic cancer in Korea. J Korean Med Sci.

